# Design of Nanosystems for the Delivery of Quorum Sensing Inhibitors: A Preliminary Study

**DOI:** 10.3390/molecules25235655

**Published:** 2020-11-30

**Authors:** Supandeep Singh Hallan, Paolo Marchetti, Daria Bortolotti, Maddalena Sguizzato, Elisabetta Esposito, Paolo Mariani, Claudio Trapella, Roberta Rizzo, Rita Cortesi

**Affiliations:** 1 Department of Chemical and Pharmaceutical Sciences, University of Ferrara, I-44121 Ferrara, Italy; hllsnd@unife.it (S.S.H.); mcp@unife.it (P.M.); brtdra@unife.it (D.B.); sgzmdl@unife.it (M.S.); trpcld@unife.it (C.T.); rbr@unife.it (R.R.); 2 Biofilms—Research Center for Biointerfaces, Faculty of Health and Society, Malmö University, SE-20506 Malmö, Sweden; 3 Biotechnology Interuniversity Consortium (C.I.B.), Ferrara Section, University of Ferrara, I-44121 Ferrara, Italy; 4 Department of Life and Environmental Sciences, Polytechnic University of Marche, I-60131 Ancona, Italy; p.mariani@staff.univpm.it

**Keywords:** nanotechnological systems, liposomes, QS inhibitors, drug delivery, biofilm, MTT test

## Abstract

Biofilm production is regulated by the Quorum Sensing system. Nowadays, Quorum Sensing represents an appealing target to design new compounds to increase antibiotics effects and avoid development of antibiotics multiresistance. In this research the use of liposomes to target two novel synthetic biofilm inhibitors is presented, focusing on a preformulation study to select a liposome composition for in vitro test. Five different liposome (LP) formulations, composed of phosphatidyl choline, cholesterol and charged surfactant (2:1:1, molar ratio) have been prepared by direct hydration and extrusion. As charged surfactants dicetyl phosphate didecyldimethylammonium chloride, di isobutyl phenoxy ethyl dimethyl benzyl ammonium chloride and stearylamine (SA) and have been used. Liposome charge, size and morphology were investigated by zeta potential, photon correlation spectroscopy, small angle x-ray spectroscopy and electron microscopy. LP-SA was selected for the loading of biofilm inhibitors and subjected to high performance liquid chromatography for entrapment capacity evaluation. LP-SA loaded inhibitors showed a higher diameter (223.6 nm) as compared to unloaded ones (205.7 nm) and a dose-dependent anti-biofilm effect mainly after 48 h of treatment, while free biofilm inhibitors loose activity. In conclusion, our data supported the use of liposomes as a strategy to enhance biofilm inhibitors effect.

## 1. Introduction

Biofilms can be described as a dispersion of immobilized microbial colonies within a self-produced matrix of hydrophilic extracellular polymers such as polysaccharides, proteins and nucleic acids. Many factors such as surface properties, nutrient availability and the presence of microbial constituents are able to influence the structure and composition of biofilms [1]. Indeed, biofilm forming bacteria initially grow reversibly attached to a surface then create an irreversible attachment facilitating their survival and leading to a more aggressive and resistant host infection [2,3]. However, biofilm formation is a very complex process in which some cells, when it reaches maturity, begin to detach and separate from the aggregates repeatedly, leading to a continuous generation of bacteria able to spread and originate new microcolonies [4]. For this reason, bacterial biofilm represents a challenge in counteracting different typologies of infection, including chronic and nosocomial infections. The huge and inappropriate use of antibiotics during the last four decades have reduced the efficacy of traditional antibiotics increasing the rising of new bacterial defense mechanisms, together with the spread of antimicrobial resistance. To date in the European Union the infections associated with biofilms and antimicrobial resistance (AMR) are a leading cause of morbidity and of increased healthcare costs [5]. Hence, the inhibition of biofilm formation can be considered a suitable target to design new antibacterial therapies.

Biofilm formation is finely regulated by the Quorum Sensing (QS) system acting intercellular communication through molecular chemical signals that are able to control the production of virulence factors and other biofilm cellular functions [6,7,8]. Therefore, compounds able to interfere with communication systems could be able to reduce the production of virulence factors and also affect biofilm formation in order to control the development of resistant mutants. In this view, QS inhibitors have gained substantial attention to counteract bacterial infection [9], in order to increase the susceptibility to antibiotics and the clearance of the pathogen by the host immune system [10]. One of the most studied QS system is that belonging to Pseudomonas aeruginosa (*P. aeruginosa*), a Gram negative bacterium often associated to chronic lung diseases (e.g., cystic fibrosis) and nosocomial infections, that takes advantage by biofilm formation to avoid both the host immune system and antibiotics effect [11,12]. *P. aeruginosa* possesses two QS systems, Las and Rhl characterized by a hierarchical structure (with Las as the main QS system) and include intracellular receptors that bind specific soluble lactones and function as transcriptional factors [13,14]. The Las system is controlled by the transcriptional activator LasR and the autoinducer synthase enzyme LasI [15]. Recently, in our department, two novel synthetic QS inhibitor (QSi) have been synthesized, namely CDC and PF based on a cyclopentilamine ring (i.e., homoserine lactone analogues) in order to mimic the N-(3-oxododecanoyl)-L-homoserine lactone (3OC12-HSL) scaffold involved in the synthesis of virulence factors and biofilm formation [15].

A critical point in the treatment of biofilm-based bacterial infections is the ability of QSi to efficiently overcome the mucous biofilm layer. In order to bypass this drawback both allowing the transport of effective concentration of drug through biofilm and a controlled release, the possibility to use liposomes was investigated. Particularly, this preformulation study focuses on the selection of a liposome composition suitable for new inhibitors of *P. aeruginosa* biofilm formation to be tested in vitro.

Among the different drug delivery technologies developed for treatment of the biofilm-related infections, nanotechnology plays an important role. Notably, a variety of nano-scaled drug delivery systems have been designed and employed, including polymeric nanoparticles, dendrimers, metal nanoparticles, solid lipid nanoparticles and liposomes [16,17,18,19,20]. Notably, the use of drug delivery systems such as liposomes is a pharmaceutical formulation considered to manage effective to achieve a therapeutic effect in humans. As reported in literature, liposomes can be considered one among different nanoparticle platforms, used to facilitate the delivery of antimicrobials to the infection site [20]. For instance, the use of liposomes as drug carriers seems to be advantageous over other delivery platforms used to prevent biofilm formation on biomedical surfaces, as demonstrated by the number of liposomal formulations approved for clinical use for this purpose at the end of the first decade of this century [21]. Therefore, these systems already suitable for conventional antibiotic delivery, should be useful for delivery of QSi formulations too.

Liposomes are the most used vesicles to control drug delivery, due to their composition similar to the eukaryotic and prokaryotic cell membrane. Liposomes are nano-sized to microsized spherical structures consisting of two layers of naturally or synthetic phospholipids, such as phosphatidylcholine, phosphatidylethanolamine, phosphatidylserine or phosphatidylglycerol surrounding an aqueous core. The liposome-forming lipid bilayer identifies two different sections: A lipophilic compartment and an inner hydrophilic core. Due to its characteristics, the core encapsulates water-soluble molecules while the hydrophobic domain of the bilayer is responsible for entrapping lipophilic agents. Liposomes characteristics, including size, lamellarity and encapsulation efficacy and positive or negative charge, are influenced either by the lipid composition and the preparation method used [22]. Cationic liposomes can be prepared by incorporating cationic surfactant in the bilayers, therefore allowing a high-yield interaction with negative charged molecules.

Summarizing, in the present paper the synthesis of two new QSi followed by the design and production by direct hydration and extrusion of five different liposomes formulations composed of phosphatidyl choline (PC), cholesterol (CH) and charged surfactant (CS) is described. Liposomes have been then characterized in terms of size, charge, morphology and in vitro activity to select the more suitable composition for the loading of the two novel biofilm inhibitors. The selected liposome composition has been then studied in terms of morphology, size, entrapment capacity and activity in vitro on biofilm and compared to the activity of the corresponding free compound.

## 2. Results and Discussion

### 2.1. Synthesis and Activity of LasR Antagonist

**Figure 1:**
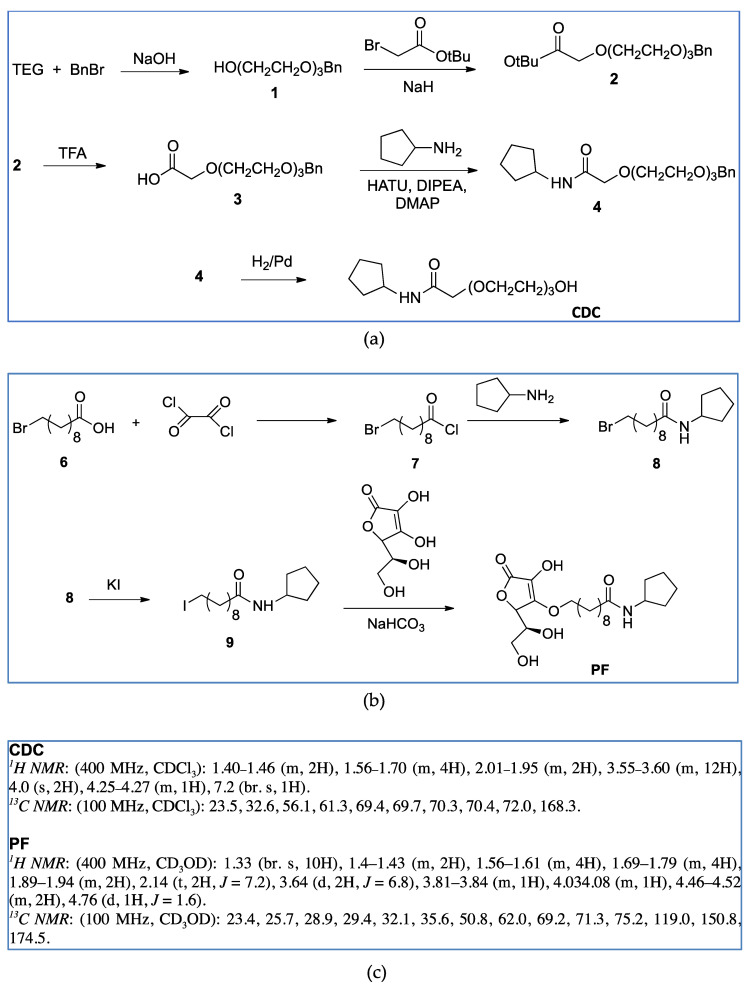
Synthetic flow charts for CDC (**a**) and PF (**b**). ^1^H and ^13^C NMR spectra values of final products (**c**).

On the basis of our previous results [12], two compounds with molecular features characterized by high probability to act efficiently as QSi were synthetized, namely CDC and PF (Table 1, Figure 1). The compounds were synthetized as described in the Materials and Methods section following the synthetic flow schemes reported in Figure 1. Moreover, the description of the synthesis and characterization of every intermediate molecule obtained in each synthetic step are reported in the Supplementary Materials.

QSi are based on the 3-oxoacyl-beta-keto-amide structure containing a polyoxyethylene chain and a cyclopentylamine to increase both stability and solubility in aqueous medium. In addition, PF compound also has ascorbic acid (vitamin C) in order to take advantage by its natural antimicrobic function to enhance the inhibitory effect on the biofilm. Since these compounds are designed to antagonize LasR receptor, which functions as a transcriptional factor, they act at cytoplasmic levels. Moreover, as above underlined, liposomes can be used to facilitate the delivery of antimicrobials to the infection site differently from others nanoparticle platforms [20,23].

**Figure 2:**
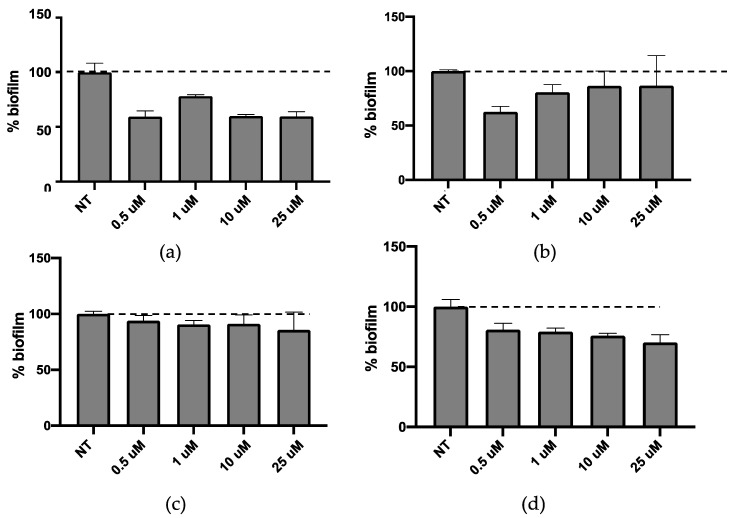
Activity on biofilm of CDC (**a**,**c**) or PF (**b**,**d**) after 24 h (**a**,**b**) and 48 h (**c**,**d**) of treatment.

QSi are not antibiotics, but molecules able to reduce biofilm formation, therefore CDC and PF were assessed for their ability to inhibit biofilm formation. Both molecules were tested in the concentration range between 0.5 and 25 μM for 24 h and 48 h on bacterial growth by measuring the bacterial OD at 600 nm [12] and the obtained results indicated that they did not affect the growth of *P. aeruginosa* (data not shown). Afterwards, the effect of both QSi was tested on biofilm formation by colorimetric biofilm assay. As described in Figure 2, it was observed that after 24 h of treatment CDC showed significant biofilm inhibition at 0.5 and 1 µM (*p* < 0.01) (Figure 2a), while all the concentrations tested for PF are able to significantly inhibit *P. aeruginosa* biofilm formation in a range comprised between 20–40% (*p* < 0.01) (Figure 2b). On the other hand, after 48 h of treatment both CDC (Figure 2c) and PF (Figure 2d) decreased their inhibitory effect on biofilm. These data suggest that the amount of QSi added to bacteria cultures was enough to counteract the quantity of QS molecules produced during a 24 h culture, not of the following 48 h.

### 2.2. Preformulatory Study: Liposome Preparation and Characterization

In order to find a formulation able to allow the efficient passage of QSi through the mucous biofilm layer, the use of liposomes was investigated by conducting a preformulation study to select the more suitable liposome composition for in vitro test. Particularly, liposomes composed of PC, CH and CS in mixture 2:1:1 (molar ratio), respectively, have been prepared by direct hydration. Charged liposomes were prepared alternatively by adding to the PC/CH mixture the following CS, namely dicetyl phosphate (LP-DCP), didecyldimethylammonium chloride (LP-DDAC), di isobutyl phenoxy ethyl dimethyl benzyl ammonium chloride (LP-DEBDA) and stearylamine (LP-SA). As a comparison, plain liposomes constituted by PC/CH (LP-P) were also prepared. After preparation, extrusion was performed to obtain unilamellar vesicles with a homogeneous size distribution [24]. This strategy allowed the reduction of vesicles mean diameters up to three-fold as compared to their size after production and at the meantime decreasing the polydispersity and improving the size distribution (data not shown) [24].

**Figure 3:**
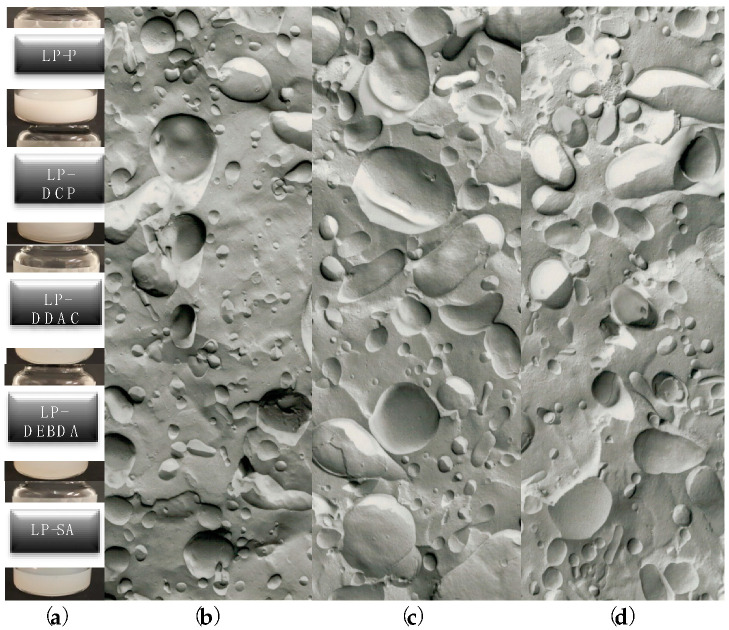
(**a**) macroscopic aspect of plain liposomes constituted by phosphatidyl choline/cholesterol (LP-P), dicetyl phosphate (LP-DCP), didecyldimethylammonium chloride (LP-DDAC), di isobutyl phenoxy ethyl dimethyl benzyl ammonium chloride (LP-DEBDA) and stearylamine (LP-SA) and representative electron microphotographs of (**b**) plain (LP-P), (**c**) anionic (LP-DCP) and (**d**) cationic (LP-DDAC) liposomes.

Extruded-liposomes showed similar macroscopic aspects, characterized by homogeneous slightly white dispersions (see Figure 3a), even if plain LP-P appeared less transparent as compared to charged formulations LP-DCP, LP-DDAC, LP-DEBDA and LP-SA (Figure 3a). From the morphological point of view, each category of liposomes was visualized by mean of freeze-fracture electron microscopy. A representative series of electron microphotographs are reported in Figure 3b–d showing plain LP-P, anionic LP-DCP and cationic LP-DDAC liposome formulations, respectively. The three categories of liposomes displayed unilamellar morphology even if a certain grade of polydispersity was evident. These results corroborated the extruded dimensional data of the five formulations obtained by photon correlation spectroscopy (PCS) analyses performed the same day of production and reported in Table 2.

All the formulations showed vesicles mean diameters around 200 nm, ranging from 180 to 205 nm with a slight increase in the case of LP-SA. Concerning the polydispersity indexes, a slight increase was evident in the presence of CS in the formulation, as compared to plain liposomes. However, a monomodal distribution was appreciable, since P.I. values were below 0.4.

The zeta potential values contributed to a good stability of liposome formulations during time, especially significant upon addition of CS (data not shown). Indeed, thanks to their polar intercalation within the phospholipid bilayer, CS confer charge to the vesicles, increasing zeta potential values with respect to plain liposomes, therefore promoting their physical stability over time by mean of charge repulsion.

**Figure 4:**
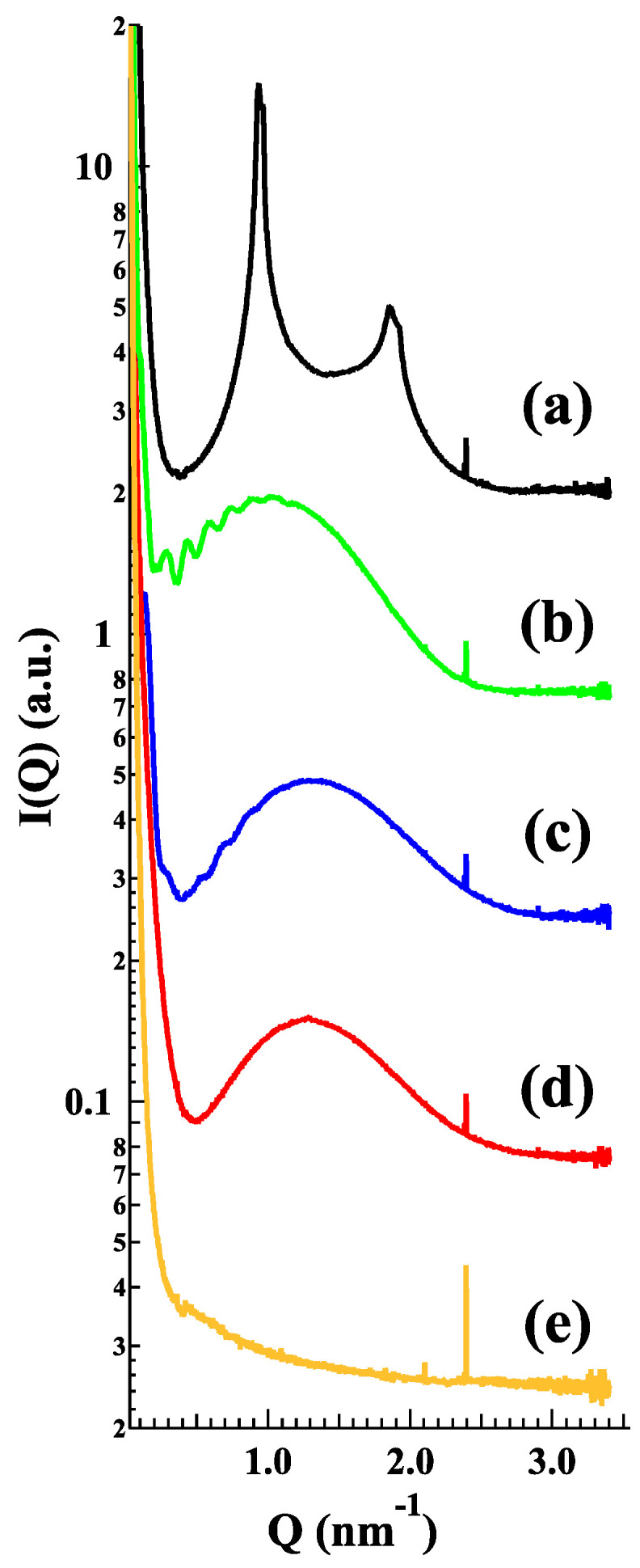
Small-angle X-ray scattering (SAXS) profiles of the different empty LP-P (**a**), LP-DDAC (**b**), LP-DEBDA (**c**), LP-SA (**d**) and LP-DCP (**e**). Experiments were performed at Diamond Light Source (UK).

In order to shed light on the structural characteristics of the produced empty liposomes, LP-P, LP-DPC, LP-DDAC, LP-DEBDA and LP-SA were investigated by small-angle X-ray scattering (SAXS). Results related to the effect of cationic and anionic surfactants on unloaded PC liposomes are reported in Figure 4. In particular, plain liposomes LP-P (Figure 4a) showed the common small angle X-ray diffraction pattern of multilamellar vesicles, characterized by a bilayer repeat distance (which measures the sum of the bilayer thickness and the thickness of the water layer separating two adjacent bilayers) of about 6.72 nm [24,25]. In contrast, the patterns b–e (Figure 4) demonstrate that the addition of CS to liposome composition leads to a loss of positional correlations between adjacent bilayers, probably due to surface charge density conferred to the bilayers upon surfactant insertion. Indeed, the X-ray diffraction profiles of LP-DDAC, LP-DEBDA and LP-SA display a broad band centered at about 0.14 Å^−1^ without any trace of diffraction peaks, e.g., a typical bilayer form factor scattering pattern indicative of the formation of unilamellar vesicles [25,26]. The effect is even greater for anionic surfactants in which the absence of scattering signal observed in the case of LP-DCP suggests a complete loss of structure. A last point should be noticed as the position of the broad band roughly corresponded to the bilayer thickness: The thickness is about 4.7 nm for LP-SA and LP-DEBDA, but increased to about 5.5 nm when liposomes have been prepared in the presence of DDAC. Such an effect could be related to changes induced by DDAC in PC lateral interactions.

### 2.3. Selection of the Liposome Formulation for LasR Antagonist Loading

**Figure 5:**
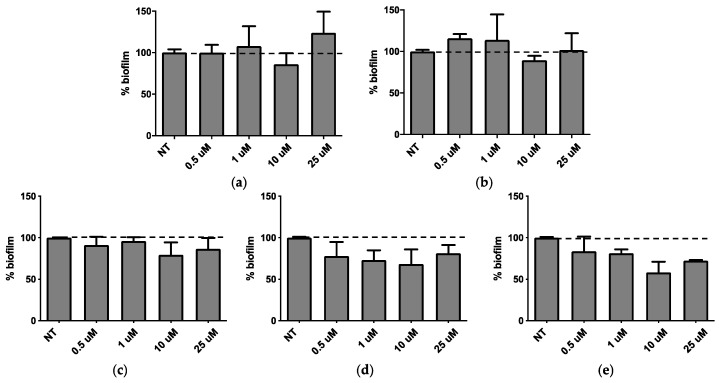
Activity of empty liposomes on biofilm after 24 h of treatment. (**a**) LP-P, (**b**) LP-DCP, (**c**) LP-DDAC, (**d**) LP-DEBDA and (**e**) LP-SA.

In order to allow the selection of the optimal liposome composition for biofilm inhibitors loading, the five empty liposome formulations were subjected to different in vitro assays, to test their activity on biofilm formation and viability on *P. aeruginosa* cultures and A594 pulmonary cells, respectively. In view to propose this system as a topic treatment by inhalatory administration, A549 lung cell line was selected to possibly mimic what occurs in vivo. Indeed *P. aeruginosa* infection often affects the airway system and mainly interacts with epithelial lung cells. Moreover, chronicization of *P. aeruginosa* infection in the lungs is involved in airway diseases (e.g., BCPO and cystic fibrosis). *P. aeruginosa* cultures were treated for 24 h with a liposome concentration ranging from 0.5 to 25 µM (Figure 5). None of the liposome formulations affected the bacterial growth at all tested concentrations (data not shown). It was found that LP-P and LP-DCP liposomes induced biofilm formation (Figure 5a,b). On the other hand LP-DDAC and LP-DEBDA slightly reduced biofilm formation (Figure 5c,d), while LP-SA liposome significantly inhibited biofilm formation at concentrations of 10 and 25 µM (Figure 5e, *p* < 0.01). We obtained similar results with a 48 h treatment (data not shown).

**Figure 6:**
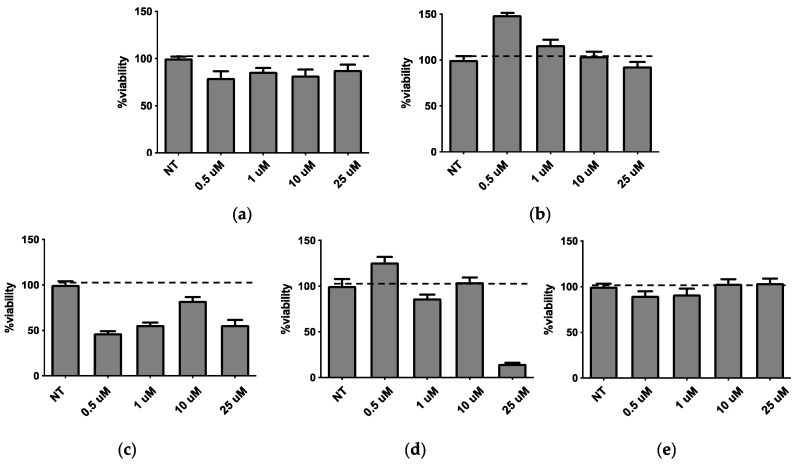
Effect on A549 pulmonary cells viability (MTT assay) after 24h of treatment with LP-P (**a**), LP-DCP (**b**), LP-DDAC (**c**), LP-DEBDA (**d**) and LP-SA (**e**).

Concerning the pulmonary A594 viability performed using MTT test, the obtained results are reported in Figure 6. It was found that plain LP-P (Figure 6a) and anionic LP-DCP (Figure 6b) showed a slight or no cytotoxicity on pulmonary cells. On the other hand, cationic liposome formulations demonstrated a different behavior, depending on the type of cationic surfactant used. Notably, LP-DDAC (Figure 6c) and LP-DEBDA (Figure 6d) showed a strong cytotoxic effect, whilst LP-SA (Figure 6e) displayed no cytotoxicity.

Taking together the effect of empty liposomes on cell viability, the bacterial growth and biofilm formation with the results obtained by preformulation study concerning the characterization of empty liposomes (i.e., homogeneous aspect, monodisperse distribution and unilamellar morphology), LP-SA has been selected as composition suitable for the loading of the new synthesized biofilm inhibitors CDC and PF.

### 2.4. Loading of Biofilm Inhibitors on LP-SA Liposomes

LP-SA containing biofilm inhibitors CDC (LP-SA/CDC) and PF (LP-SA/PF) were prepared by direct hydration and extrusion using the same scheme procedure as described for unloaded formulations. However, QSi were dissolved in aforementioned organic solvents along with other components to achieve final concentration 10 mM in the dispersion.

The entrapment capacity (EC) of each drug within LP-SA was determined after 1 day from liposome production, as described in the Materials and Methods section. Namely, loaded LP-SA dispersion was separated with a filter centrifugation and an amount of supernatant with loaded liposomes was disaggregated and quantified by HPLC for drug content [22].

The obtained results summarized in Table 3, show that both compounds were quantitatively loaded within the vesicles. Concerning the effect of the loading onto the vesicle size, it was found that both QSi displayed an increase of the mean size of liposomes, being around 11% for CDC and 20% for PF, but maintained a quite homogeneous size distribution, as indicated by polydispersity index values (Table 3).

**Figure 7:**
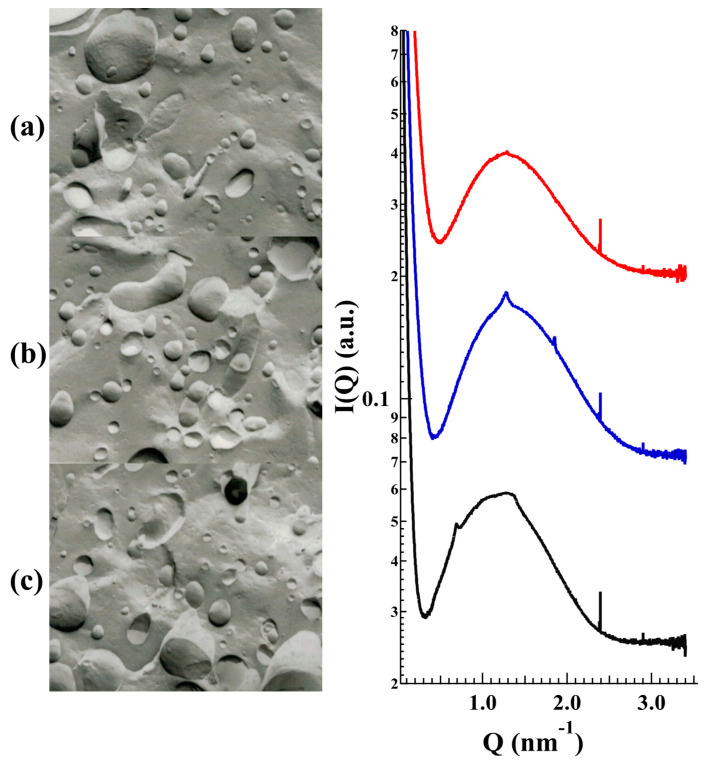
Freeze-fracture electron microphotographs (left) and SAXS profiles (right) of LP-SA formulations either unloaded (**a**, red) or CDC (**b**, blue) and PF (**c**, black) loaded. SAXS experiments were performed at Diamond Light Source (UK).

Regarding influences of QSi on deep liposome morphology, Figure 7 reports the freeze-fracture electron microphotographs (left) and the SAXS profiles (right) obtained from unloaded and CDC or PF loaded LP-SA. The scattering pattern is essentially characterized by the bilayer form factor, but the drug presence induced a low-intensity diffraction pattern, which possibly corresponded to a multilamellar structure characterized by a low positional correlation between adjacent bilayers and/or by a very low number of stacked bilayers [26]. The measured repeat distances (10.05 and 9.20 nm for CDC and PF, respectively) displayed a larger value as compared to that observed in plain PC/CH liposomes (6.72 nm), therefore confirming strong repulsive bilayer-bilayer interactions induced by the charged surfactants on the PC bilayers.

### 2.5. In Vitro Experiments of LP-SA Loaded Biofilm Inhibitors

**Figure 8:**
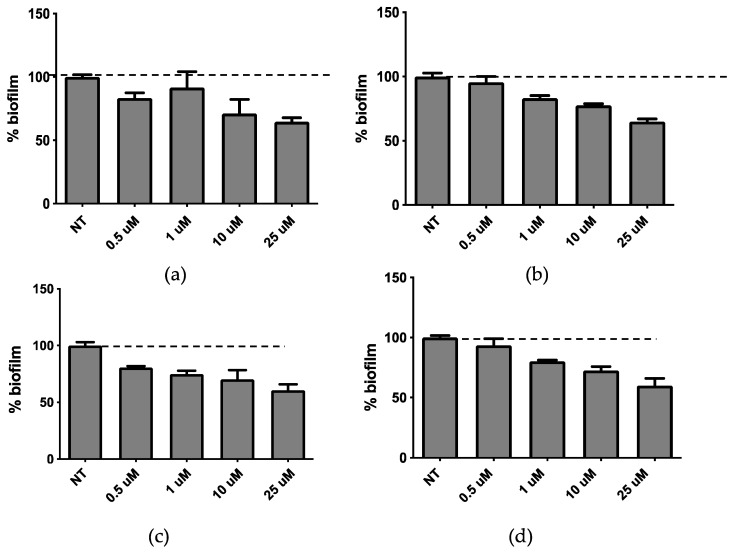
Activity on biofilm of LP-SA loaded CDC (**a**,**c**) or PF (**b**,**d**) after 24 h (**a**,**b**) and 48 h (**c**,**d**) of treatment.

The release of QSi from LP-SA formulations was determined by dialysis as described in the Supplementary Materials. The results reported in Figure S1 indicated that both CDC and PF are released in a controlled manner reaching after 28 h at least the 60% and 40% of total amount of QSi, respectively for CDC and PF. QSi-loaded LP-SA were then assessed for biofilm inhibitory activity. The liposome formulation did not affect bacterial growth after 24 h of treatment at the concentration range between 0.5–25 µM (data not showed). The biofilm assay performed at the same experimental conditions showed the inhibition of biofilm formation for both compounds in a dose-dependent manner (Figure 8a,b), that was significant for concentrations above 10 µM and 1 µM for liposomes carrying CDC and PF, respectively (*p* < 0.01). The liposome formulation of both compounds led to a dose-dependent effect on biofilm formation.

When we compared the results obtained on biofilm inhibition with the QSi alone (Figure 1) or liposome-loaded (Figure 8), we observed an effective increased biofilm inhibition for LP-SA/CDC 10 and 25 µM as compared to the same concentration of sole CDC. It was hypothesized that the loading of QSi compounds into LP-SA might reduce the release of the active compounds and needed a longer treatment. Therefore, biofilm inhibition experiments in the presence of sole or LP-SA loaded QSi, have been repeated extending the treatment to 48 h. Particularly, after 48 h of treatment both CDC (Figure 8c) and PF (Figure 8d) decreased their inhibitory effect on biofilm when used alone as compared to the 24 h treatment (Figure 8a,b). On the contrary, when both QSi were administered as liposomal formulation, they maintained and increased their effect on biofilm formation (Figure 4c,d), showing their activity already at low concentrations (LP-SA/CDC: 0.5 µM, *p* = 0.0005; LP-SA/PF: 1 µM, *p* < 0.0001). Therefore, it can be evidenced that QSi-loaded liposomes enhanced their ability to interfere with biofilm formation supporting a longer activity as compared to the free form of each QSi up to 48 h from the treatment. These data confirm the hypothesis of a gradual release of QSi from the liposomes, therefore enabling to maintain and to enhance the treatment efficacy for a longer period. This effect is particularly evident for CDC compound, which presented an increased effect already at the concentrations of 0.5 and 1 µM, as compared to the administration of CDC alone (0.5 µM: 81% vs. 94%; 1 µM: 75% vs. 90%; *p* < 0.01). The enhancement of this effect should be possibly ascribed to a better distribution of lipophilic molecules in aqueous environment due to the distribution within vesicle bilayer, therefore influencing its bioavailability [27].

## 3. Materials and Methods

### 3.1. Materials

Soybean lecithin (PC) (90% phosphatidylcholine) used for liposome preparation was Epikuron 200 from Lucas Meyer, Hamburg, Germany. CH, DCP, DDAC, DEBDA and stearylamine (SA) were purchased from Sigma-Aldrich (St Louis, MO, USA). Solvents and all other chemicals were of analytical grade and were from Merck Serono S.p.A. (Rome, Italy).

### 3.2. Synthesis and Characterization of Las QS Inhibitors

Synthetic flow schemes of CDC and PF are reported in Figure 1. Commercially available reagents were used without further purification. NMR spectra were recorded on a Varian Mercury Plus 400 spectrometer at 400 MHz (^1^H) and 100 MHz (^13^C). Peak positions are given in parts per million (δ) down-field from tetramethylsilane used as internal standard. J values are expressed in hertz. Electrospray mass spectra were recorded on a Waters Micromass ZQ-20000 instrument. All reactions were monitored by thin-layer chromatography and/or reversed-phase high-performance liquid chromatography (HPLC). Analytical TLC was carried out using Merck precoated silica gel F-254 plates. Preparative flash chromatography was done using Merck silica gel 60 (0.063–0.200 mm) using the indicated eluent. Solvents were dried according to standard procedures and reactions requiring anhydrous conditions were performed under argon atmosphere. Solutions containing final product were dried with Na_2_SO_4_, filtered and concentrated under reduced pressure using a rotatory evaporator. Analytical RP-HPLC was performed on a Beckman System Gold 168 using C18 columns, namely a Phenomex Luna (4.6 mm by 10 cm, 3 µ) for CFC and a Waters XBridge (4.6 mm by 15 cm, 5 µ) for PF, the flow was 0.5 mL/min with detection at 220 nm and with a binary eluents system (eluent A: H_2_O + 0.1% TFA, eluent B: CH_3_CN + 0.1% TFA), using a linear gradient (t = 0 min: 0% B, t = 25 min: 100% B). Final compounds were purified by preparative HPLC Water Delta Prep 4000 with detection at 220 nm using an XTerra C18 column (30 by 50 mm, 5 µ) with the binary system (eluent A: H_2_O + 0.1%TFA, eluent B: 60% CH_3_CN and 40% H^2^O + 0.1% TFA) using a linear gradient (t = 0 min: 15% B, t = 30 min: 100% B, t = 40 min: 100% B). Melting points were measured with a hot plate Reichert-Kofler microscope and are uncorrected.

### 3.3. Liposomes Preparation

Plain (PC/CH, 2:1 molar ratio) and charged (PC/CH/CS, 2:1:1 molar ratio) liposomes have been obtained using direct hydration method with slight modifications [28]. In detail, liposome dispersions were produced by solubilizing 25 mg/mL lipid phase in methylene chloride/methanol mixture (1:1, *v*/*v*). The organic solvent residue was removed using a rotary evaporator under vacuum in order to attain a thin lipid film, which was subsequently hydrated with 2 mL of sterile water followed by 10 min swirling. Liposomes were then subjected to extrusion to obtain unilamellar vesicles with a homogeneous size distribution [24]. For this, liposomal suspension was subjected to five extrusion cycles through two stacked standard 25 µm diameter polycarbonate filters with 0.2 µm pore size (Nucleopore Corp., Pleasanton, CA, USA) supported by 25 µm polyester drain disk. 10–20 bars nitrogen pressure has been maintained within an extruder (Lipex Biomembranes, Vancouver, BC, Canada) during the entire process. Finally, liposomes were collected and stored for further studies.

QS inhibitors loaded liposomes were prepared using a similar scheme. However, QS inhibitors dissolved in aforementioned organic solvents along with other components to achieve final concentration 10 mM in the system.

### 3.4. Liposome Characterization

#### 3.4.1. Freeze-Fracture Electron Microscopy

Aliquots of liposome preparations were sandwiched between copper plates and frozen using liquid propane (−180 °C). Afterwards, the holders containing the frozen samples were transferred to a Balzers BAF 400 freeze replica apparatus (Balzers, Liechtenstein) and subjected to fracture at −150 °C. The samples were immediately replicated with Pt/C (2 nm) at an angle of 45° followed by C (20 nm) at an angle of 90°. After cleaning stripped replicas with 30% sodium hypochloric and potassium dichromate-H_2_SO_4_ solution followed by distilled water, they were mounted on 300-mesh Ni grids, and visualized after drying by mean of a transmission electron microscope (JEM200 CX, JEOL, Tokyo, Japan). Micrographs were taken randomly within replica regions representative of the sample.

#### 3.4.2. Photon Correlation Spectroscopy (PCS) and Zeta Potential

Vesicle size measurements were performed on aqueous diluted liposome samples (1:20 by volume) using a Zetasizer Nano S90 (Malvern Instr., Malvern, UK) equipped with a 5 mW helium neon laser with a wavelength output of 633 nm, whist zeta potential measurements were performed using Zetasizer ultra (Malvern panalytical Ltd., Malvern, UK). Plastic-ware was cleaned with detergent washing and rinsed twice with milliQ water. Measurements were made at 25 °C at an angle of 90°, run time around 180 s. Data were interpreted by the “CONTIN” method [29].

#### 3.4.3. Small Angle X-rays Scattering (SAXS)

Small angle X-ray scattering (SAXS) experiments were performed at the bioSAXS beamline B21 in Diamond Light Source (Harwell, UK). Loaded and unloaded liposomes were put into small tubes in an automated sample changer. Samples were then moved into a temperature-controlled quartz capillary and exposed for 1 s, acquiring 30 frames at 20 °C. Data were collected utilizing a Dectris Eiger 4M (75 by 75 μm pixels) detector. The sample-detector distance was a 3.7 m and the X-ray wavelength λ = 0.1 nm, so that the explored Q-range extended from 0.026 to 3.4 nm^−1^. 2D data were corrected for background, detector efficiency and sample transmission and were radially averaged to derive I(Q) vs. Q curves [30].

### 3.5. Drug Content within Liposomes

The entrapment capacity (EC) of each drug in liposomes has been determined after 1 day from liposome production. Namely, 500 μL of drug loaded liposome dispersion were poured in a centrifugal filter (Microcon centrifugal filter unit YM-10 membrane, NMWCO 10 kDa, Sigma-Aldrich, St. Louis, MO, USA) and centrifuged at 8000 rpm for 20 min on a Spectrafuge™ 24D Digital Microcentrifuge, (Woodbridge, NJ, USA). Hundred microliters of supernatant with loaded liposomes have been diluted with ethanol (1:10, *v*/*v*) and maintained under magnetic stirring for 30 min. Afterwards the obtained solution has been filtrated through a nylon syringe filter (0.22 μm pores) and the amount of drug quantified by HPLC as below reported. The EC was determined using the following equation:

EC = QS inhibitor-d/QS inhibitor-i × 100(1)

in which QS inhibitor-d is the amount of drug detected from HPLC analysis and QS inhibitor-i is the total amount of drug used for liposome production. Measurements were conducted at least thrice for each sample and the mean values ± standard deviations were calculated [31].

Reverse phase high performance liquid chromatography (RP-HPLC) analyses of QS inhibitors containing LP-SA have been performed on a Beckman HPLC System Gold equipped with a 126 Solvent Module and a UV detector Module 168. In the case of CDC, samples have been loaded on a stainless steel Zorbax^®^ C18 (4.6 by 150 mm) packed with 3.5 μm particles (Agilent, Santa Clara, CA, USA), while for PF, samples on a stainless steel Kinetex C18 (4.6 by 150 mm) packed with 5 μm particles has been used (Phenomenex Inc., Torrance, CA, USA). The compounds have been monitored at 220 nm using a linear gradient from 0% of solvent A (water containing 0.1% TFA) to 100% solvent B (acetonitrile containing 0.1% TFA) over 25 min at a flow rate of 0.7 mL/min in the case of CDC (retention time 5.7 min) and at 0.5 mL/min flow rate in the case of PF (retention time 16.89 min). Data acquisition and processing were performed using 32Karat 8.0 software. The method was validated for linearity (R2 = 0.995), repeatability (relative standard deviation 0.02%, n = 6 injections) and limit of quantification (0.04 μg/mL). Analyses were conducted in triplicate, mean and standard deviations values were calculated.

### 3.6. Cell Culture and Cytotoxicity Studies

The effect of plain, cationic or anionic liposomes on cell proliferation was determined on in vitro cultured human lung carcinoma A549 cells (ATCC^®^ CCL-185™) [27]. Standard conditions for cell growth were D-MEM/F-12 (1:1) (1X), liquid—with L-Glutamine (Invitrogen), medium supplemented with 10% fetal calf serum (Irvine Scientific, Santa Ana, CA, USA) and Pen-Strep 1× (Omega Scientific Inc., Tarzana, CA, USA) in 5% CO2 at 90% humidity. Cell viability was determined by MTT test (3-(4,5-dimethilthiazol-2yl)-2,5-diphenyl tetrazolium bromide) colorimetric assay (Roche Diagnostics Corporation, Indianapolis, IN, USA) following the manufacturer’s instructions as previously described [32]. Cell viability was determined by treating A549 cells with different concentration of cationic nanocarriers in terms of cationic detergent, from 0 to 25 μM, for 24 h. Afterwards absorbance was read at 590 nm.

### 3.7. P. aeruginosa Culture and Biofilm Formation Assay

Pseudomonas aeruginosa PAO1 strain was grown at 37 °C in aerobic condition in M9 Minimal Salts medium (Sigma-Aldrich, St Louis, MO, USA). Biofilm studies were performed on planktonic *P. aeruginosa* PAO1 culture by microtiter plate colorimetric assay [28]. After overnight growth, optical density at 600 nm was assessed to determine bacterial growth. After appropriate dilution in M9 medium, 100 µL of *P. aeruginosa* suspension was added in each well of a microtiter plate. The optical density (OD600) at inoculation was 0.04. Bacteria were then treated with different concentration (0.1–25 µM) of QS inhibitors, empty liposomes and drug-encapsulated liposomes in triplicates for 24 h or 48 h at 37 °C. Broth without bacteria was used as negative control. Extent of biofilm formation was measured using crystal violet dye. Briefly, after incubation, the wells were washed three times with phosphate-buffered saline (PBS; Sigma-Aldrich) and plate was 60 °C-dried for 90 min. Samples were then stained with 200 μL of crystal violet solution (1%) for 15 min. After incubation, wells were washed several times with tap water to eliminate the excess of dye and the plate air-dried. Then, 100 μL of acetic acid 33% was added to each well to solubilize crystal violet and the absorbance read at 570 nm.

## 4. Conclusions

This preliminary study was finalized to evaluate the possible employment of liposomes as carriers for QSi. By combining the results obtained from the preformulation study on the choice of liposomes lipid composition with those on the cytotoxic effect and the inhibitory activity on the formation of the biofilm, it was possible to select LP-SA as a liposomal formulation to proceed with the loading of two QSi synthesized in our laboratories. It was found that both molecules could be efficiently loaded within LP-SA and that no affection on bacterial growth after 24 h of treatment at the concentration range between 0.5–25 µM occurred. Moreover, both QSi-loaded liposomes led to a dose-dependent effect on biofilm formation but an effective increased biofilm inhibition for CDC loaded LP-SA was observed as compared to the same concentration of CDC alone, especially after 48 h treatment.

In conclusion, our results suggest that LP-SA could be considered a promising delivery system for QSi administration, especially for those compounds showing a lipophilic character, probably in reason of a better dissolution and subsequent bioavailability in aqueous environment. However, studies are underway to confirm these resuts, to test the stability over time of the vescicle systems produced and to improve the release of QSi in the districts involved in the formation of biofilms.

## Figures and Tables

**Table 1:** Structure and some physico-chemical characteristics of the newly synthesized Quorum Sensing inhibitor (QSi).

Biofilm Inhibitor	Chemical Structure	MW	λmax	LogP
CDC	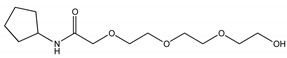	275	220	0.24
PF	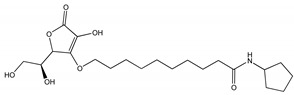	413	220	2.51

**Table 2:** Size and zeta potential of liposome formulations after production, as determined by photon correlation spectroscopy (PCS).

	LP-P (PC/CH)	LP-DCP (PC/CH/DCP)	LP-DDAC (PC/CH/DDAC)	LP-DEBDA (PC/CH/DEBDA)	LP-SA (PC/CH/SA)
Z ave (nm) ^1^	191.5 ± 1.7	180.1 ± 3.7	197.0 ± 2.1	180.1 ± 1.0	205.7 ± 1.6
P.I. ^2^	0.11 ± 0.07	0.36 ± 0.1	0.20 ± 0.08	0.36 ± 0.06	0.23 ± 0.05
ζ Potential (mV) ^3^	−28.4 ± 0.4	−67.4 ± 2.6	+90.6 ± 0.3	+92.3 ± 2.0	+63.1 ± 0.9

^1^ Zeta average diameter (mean size). ^2^ Polydispersity index. ^3^ Zeta Potential. Data are the mean of 5 independent determinations on different batches of the same dispersion ± standard deviation.

**Table 3:** Characteristics of LP-SA unloaded or loaded with biofilm inhibitors.

LP-SA	Inhibitor Concentration (mM)	Z-Ave Diameter (nm) ^1^	Polydispersity Index ^1^	ζ Potential (mV)	EC% ^1^
unloaded	/	205.7 ± 1.6	0.229 ± 0.02	+63.1 ± 0.9	/
CDC-loaded	10	223.6 ± 1.9	0.252 ± 0.01	+67.8 ± 0.4	98.8 ± 0.5
PF-loaded	10	239.6 ± 1.4	0.326 ± 0.06	+43.1 ± 1.7	99.4 ± 1.3

^1^ Values are the mean of determinations on different batches of the same type of dispersion.

## Supplementary Materials

Figure S1: Release kinetics of CDC (◯) and PF (●) from LP-SA, as determined by dialysis. Data are the mean of three experiments ± s.d.

## References

[1] Flemming H.-C., Wingender J. (2010). The biofilm matrix. Nat. Rev. Microbiol..

[2] Penesyan A., Gillings M., Paulsen I. (2015). Antibiotic Discovery: Combatting Bacterial Resistance in Cells and in Biofilm Communities. Molecules.

[3] Tacconelli E., Carrara E., Savoldi A., Harbarth S., Mendelson M., Monnet D.L., Pulcini C., Kahlmeter G., Kluytmans J., Carmeli Y. (2018). Discovery, research, and development of new antibiotics: The WHO priority list of antibiotic-resistant bacteria and tuberculosis. Lancet Infect. Dis..

[4] Hall-Stoodley L., Costerton J.W., Stoodley P. (2004). Bacterial biofilms: From the Natural environment to infectious diseases. Nat. Rev. Microbiol..

[5] European Centre for Disease Prevention and Control Homepage. https://www.ecdc.europa.eu/en.

[6] Greenberg E.P. (2003). Bacterial communication and group behavior. J. Clin. Investig..

[7] Li Y.-H., Tian X. (2012). Quorum Sensing and Bacterial Social Interactions in Biofilms. Sensors.

[8] Hentzer M., Givskov M. (2003). Pharmacological inhibition of quorum sensing for the treatment of chronic bacterial infections. J. Clin. Investig..

[9] Rasko D.A., Sperandio V. (2010). Anti-virulence strategies to combat bacteria-mediated disease. Nat. Rev. Drug Discov..

[10] Bjarnsholt T., Givskov M. (2007). Quorum-sensing blockade as a strategy for enhancing host defences against bacterial pathogens. Philos. Trans. R. Soc. B.

[11] Rizzo R., Bergamini G., Bortolotti D., Leal T., D’Orazio C., Pintani E., Melchiorri L., Zavatti E., Assael B.M., Sorio C. (2016). HLA-G expression and regulation during *Pseudomonas aeruginosa* infection in cystic fibrosis patients. Future Microbiol..

[12] Bortolotti D., Trapella C., Bragonzi A., Marchetti P., Zanirato V., Alogna A., Gentili V., Cervellati C., Valacchi G., Sicolo M. (2019). Conjugation of LasR Quorum-Sensing Inhibitors with Ciprofloxacin Decreases the Antibiotic Tolerance of *P. aeruginosa* Clinical Strains. J. Chem..

[13] Gorgani N., Ahlbrand S., Patterson A., Pourmand N. (2009). Detection of point mutations associated with antibiotic resistance in Pseudomonas aeruginosa. Int. J. Antimicrob. Agents.

[14] Lee J., Zhang L. (2015). The hierarchy quorum sensing network in Pseudomonas aeruginosa. Protein Cell.

[15] Malik A., Afaq S., Shahid M., Akhtar K., Assiri A. (2011). Influence of ellagic acid on prostate cancer cell proliferation: A caspase-dependent pathway. Asian Pac. J. Trop. Med..

[16] Guzman M., Dille J., Godet S. (2012). Synthesis and antibacterial activity of silver nanoparticles against gram-positive and gram-negative bacteria. Nanomed. Nanotechnol. Biol. Med..

[17] Xie J., Ji Y., Xue W., Ma D., Hu Y. (2018). Hyaluronic acid-containing ethosomes as a potential carrier for transdermal drug delivery. Colloids Surf. B Biointerfaces.

[18] Ma H., Williams P.L., Diamond S.A. (2013). Ecotoxicity of manufactured ZnO nanoparticles—A review. Environ. Pollut..

[19] Drulis-Kawa Z., Dorotkiewicz-Jach A. (2010). Liposomes as delivery systems for antibiotics. Int. J. Pharm..

[20] Zhang L., Pornpattananangkul D., Hu C.-M., Huang C.-M. (2010). Development of Nanoparticles for Antimicrobial Drug Delivery. Curr. Med. Chem..

[21] Sousa C., Botelho C., Oliveira R. (2011). Nanotechnology applied to medical biofilms control. Science Against Microbial Pathogens: Communicating Current Research and Technological Advances.

[22] Pattni B.S., Chupin V.V., Torchilin V.P. (2015). New Developments in Liposomal Drug Delivery. Chem. Rev..

[23] Li J., Anraku Y., Kataoka K. (2020). Self-Boosting Catalytic Nanoreactors Integrated with Triggerable Crosslinking Membrane Networks for Initiation of Immunogenic Cell Death by Pyroptosis. Angew. Chem. Int. Ed..

[24] Cortesi R., Esposito E., Cuccu I., Romagnoli R., Menegatti E., Zaid A.N., Nastruzzi C. (2007). Liposomes and Micellar Dispersions For Delivery of Benzoheterocyclic Derivatives of Distamycin A. Drug Deliv..

[25] Hodzic A., Zoumpoulakis P., Pabst G., Mavromoustakos T., Rappolt M. (2012). Losartan’s affinity to fluid bilayers modulates lipid–cholesterol interactions. Phys. Chem. Chem. Phys..

[26] Andreozzi P., Funari S.S., La Mesa C., Mariani P., Ortore M.G., Sinibaldi R., Spinozzi F. (2010). Multi- to unilamellar transitions in catanionic vesicles. J. Phys. Chem. B.

[27] Fahr A., van Hoogevest P., May S., Bergstrand N., Leigh S.M.L. (2005). Transfer of lipophilic drugs between liposomal membranes and biological interfaces: Consequences for drug delivery. Eur. J. Pharm. Sci..

[28] Puglia C., Bonina F., Rizza L., Cortesi R., Merlotti E., Drechsler M., Mariani P., Contado C., Ravani L., Esposito E. (2010). Evaluation of Percutaneous Absorption of Naproxen from Different Liposomal Formulations. J. Pharm. Sci..

[29] Pecora R. (2000). Dynamic Light Scattering Measurement of Nanometer Particles in Liquids. J. Nanopart. Res..

[30] Barbosa L.R.S., Ortore M.G., Spinozzi F., Mariani P., Bernstorff S., Itri R. (2010). The Importance of Protein-Protein Interactions on the pH-Induced Conformational Changes of Bovine Serum Albumin: A Small-Angle X-ray Scattering Study. Biophys. J..

[31] Cortesi R., Esposito E., Maietti A., Menegatti E., Nastruzzi C. (1998). Production and antiproliferative activity of liposomes containing the antitumour drug chromomycin A_3_. J. Microencapsul..

[32] Bortolotti D., LeMaoult J., Trapella C., Di Luca D., Carosella E.D., Rizzo R. (2015). Pseudomonas aeruginosa Quorum Sensing Molecule *N*-(3-Oxododecanoyl)-l-Homoserine-Lactone Induces HLA-G Expression in Human Immune Cells. Infect. Immun..

