# Supplementary material for: Design of Nanosystems for the Delivery of Quorum Sensing Inhibitors: A Preliminary Study
Source: Molecules. 2020 Nov 30;25(23):5655. doi: 10.3390/molecules25235655 (PMC7730761; doi:10.3390/molecules25235655)
Supplement: Supplementary file 1 [file molecules-25-05655-s001.pdf]

molecules

MDPI

Supplementary material

Synthesis and characteristics of intermediate 1: A solution of TEG (53.6 mL, 0.4 M), benzyl-bromide (11.9 mL, 0.1 M) and NaOH 50% (32.0 mL, 0.4 M) was refluxed for 24 h and then the reaction mixture was, in the order, diluted with H_2O (200 mL), extracted with Et2O (4 × 90 mL), dried over anhydrous Na_2SO_4 and concentrated in vacuo. Mono-protected compound 1 was isolated by distillation (T = 160 °C, P = 5 × 10^{-2} mBar). Colorless oil (71%). Rf: (CH_2Cl_2/MeOH 10/1) 0.4. MS (ESI) [M+H]+ = 241.38 ^{1}H NMR (400 MHz, CDCl3): 2.18 (s, 1H), 3.63–3.74 (m, 12H), 4.59 (s, 2H), 7.36–7.37 (m, 5H). ^{13}C NMR (100 MHz, $CDCl_3$): 61.8, 69.4, 70.5, 70.7, 70.7, 72.6, 73.4, 127.7, 127.9, 128.5, 138.2.

Synthesis and characteristics of intermediate 2: A solution of intermediate 1 (1.63 g, 6.76 mM) in anhydrous THF were added, drop by drop, to a stirred suspension of NaH 60% (0.541 g, 13.5 mM) in anhydrous THF (15 mL) in ice bath. After 30 min, tert-butyl bromoacetate (9.82 mL, 66.49 mM), was added drop by drop, and the mixture warmed to room temperature for 2 h. The pH of the mixture was then adjusted to 6 with NH_4Cl saturated solution and, after concentration to eliminate THF, the aqueous solution was extracted with ethyl-acetate (3 × 10 mL). After concentration in vacuo, the combined organic extracts were distilled in order to eliminate the excess of tert-butyl bromoacetate (T = 140 °C, P = 5 × 10^{-2} mBar). Colorless oil (76%). Rf: (AcOEt/Pet 1/2) 0.44. MS (ESI): [M+H]+ = 355, 299.47. ^{1}H NMR (400 MHz, $CDCl_3$): 1.48 (s, 9H), 3.68-3.72 (m, 12H) 4.03 (s, 2H), 4.58 (s, 2H), 7.35–7.36 (m, 5H). ^{13}C NMR (100 MHz, $CDCl_3$): 28.2, 69.1, 69.5, 70.7, 70.8, 73.3, 81.6, 110.1, 127.7, 127.8, 128.4, 138.3, 150.4, 169.8.

Synthesis and characteristics of intermediate 3: Compound 2 was dissolved in TFA (1 mL/mM of 2) and the solution magnetically stirred during 2 h. The reaction mixture was evaporated in vacuo to give 3 as colorless oil (100%). Rf: (CH_2Cl_2/MeOH 10/1) 0.1. MS (ESI): [M+H]+ = 299. ^{1}H NMR (400 MHz, $CDCl_3$): 3.67–3.71 (m, 12H), 4.16 (s, 2H), 4.60 (s, 2H), 7.28–7.35 (m, 5H), 7.36 (s, 1H). ^{13}C NMR (100 MHz, $CDCl_3$): 69.1, 69.3, 70.3, 70.4, 70.6, 70.7, 7158, 73.4, 127.9, 128.1, 128.5, 137.6, 172.8.

Synthesis and characteristics of intermediate 4: To a solution of compound 3 (3.5 g, 11.70 mM) and cyclopentylamine (1.73 mL, 17.55 mM) in DMF (20 mL), DIPEA (6.01 mL, 35.11 mM), DMAP (0.28 g, 2.34 mM) and HATU (6.67 g, 17.55 mM) were added, in the order, under magnetic stirring. After 16 h, the mixture was evaporated in vacuo and the residue diluted with ethyl-acetate (35 mL). The organic phase was washed, in the order, with HCl 2.5 N (2 × 10 mL), H_2O (2 × 10 mL), saturated solution of $NaHCO_3$ (2 × 10 mL), H_2O (2 × 10 mL) and organic solvent removed in vacuo to obtain a residue that was purified by flash chromatography. Colorless oil (55%). Rf: (CH_2Cl_2/MeOH 10/1) 0.3 MS (ESI): [M+H]+ = 366. ^{1}H NMR: (400 MHz, $CDCl_3$): 1.43–1.44 (m, 2H), 1.60–1.62 (m, 2H), 1.67–1.69 (m, 2H), 3.61–3.65 (m, 12H), 3.9 (s, 2H), 4.23–4.25 (m, 1H), 4.6 (s, 2H), 6.75 (s, 1H), 7.25–7.33 (m, 5H). ^{13}C NMR: (100 MHz, $CDCl_3$): 23.5, 32.6, 56.1, 69.4, 69.7, 70.0, 70.4, 72.0, 73.0, 127.4, 127.8, 128.6, 137.5, 168.3.

Synthesis of CDC: A suspension of compound 4 (1.53 g, 4.20 mM) and palladium on activated charcoal (2 spatula tips) in absolute EtOH was placed under H_2 atmosphere under magnetic stirring. After compound 4 disappeared on TLC (3 h), the suspension was filtered over Celite and the solvent removed in vacuum to give 5 as colorless oil (72%). Rf (CH_2Cl_2/MeOH/Tol 17/1/2) 0.2. MS (ESI): $[M+H]^+$ = 276.

Synthesis and characteristics of intermediate 7: To a solution of 10-bromodecanoic acid (0.5 g, 1.99 mM) in CH_2Cl_2 (5 mL), oxalyl chloride (0.176 mL, 2.10 mM) and DMF (3 drops) were added. After 4 h the mixture was evaporated to give compound 7 as colorless oil that was used without any purification and characterization.

Synthesis and characteristics of intermediate 8: To a solution of cyclopentylamine (0.315 g, 3.71 mM) in CH_2Cl_2 (10 mL) cooled at 0 °C and under magnetic stirring, a solution of compound 7 (0.5 g, 1.85 mM) in CH_2Cl_2 (5 mL) were added drop to drop. The reaction was allowed to warm to room temperature overnight, diluted with CH_2Cl_2 (50 mL) and washed, in the order, with HCl 2N (2 × 7 mL), brine (2 × 5 mL), saturated solution of $NaHCO_3$ (2 × 7 mL), brine (2 × 5 mL). Concentration in vacuo of organic phase gave compound 8 as colorless oil (94%). Rf: (AcOEt/Pet 1/1) 0.5. MS (ESI): [M

+ H]+ = 318. ^{1}H NMR: (400 MHz, $CDCl_3$): 1.20–1.36 (m, 10H), 1.55–1.60 (m, 8H), 1.70–1.80 (m, 2H), 1.90–1.95 (m, 2H), 2.10 (t, 2H, J = 7.1 Hz), 3.05 (t, 2H, J = 6.9 Hz), 4.10–4.22 (m, 1H), 5.40 (br. s, 1H). ^{13}C NMR: (100 MHz, $CDCl_3$): 7.2, 23.4, 25.4, 28.0, 29.0, 30.2, 33.1, 33.3, 36.5, 51.0, 172.5.

Synthesis and characteristics of intermediate 9: To a solution of compound 8 in acetone (8 mL) KI (0.498 g, 3 mM) was added and the resulting suspension refluxed during 6 h. After filtration over Celite, the solvent was removed in vacuum to give a crude yellow solid that was purified by flash chromatography (AcOEt/Pet 1/1 0.5). Light yellow crystals (98%). Pf: 81–83 °C. MS (ESI): [M + H]+ = 366 ^{1}H NMR: (400 MHz, $CDCl_3$): 1.25–1.41 (m, 10H), 1.57–1.66 (m, 8H), 1.76–1.84 (m, 2H), 1.95–2.01 (m, 2H), 2.12 (t, 2H, J = 7.2), 3.17 (t, 2H, J = 6.8), 4.16–4.22 (m, 1H), 5.42 (br. s, 1H). ^{13}C NMR: (100 MHz, DMSO): 7.4, 23.7, 25.9, 28.4, 29.2, 30.4, 33.2, 33.5, 36.9, 51.1, 172.7.

Synthesis of PF: To a solution of compound 9 (0.431 g, 1.45 mM) in DMSO (6 mL), ascorbic acid (0.635 g, 3.61 mM) and $NaHCO_3$ (0.487 g, 5.8 mM) were added and the mixture warmed at 60 °C during 3 h. The solvent was removed by distillation (T = 70 °C, P = 5 × 10^{-2} mBar) and the resulting brown oil was first diluted with ethanol (5 mL) and brine (6 mL) and then adjusted the pH till 5 with HCl 2 N. Compound PF was extracted from the mixture with ethyl-acetate (3 × 15 mL) and, after removing solvent in vacuo, the final product was purified by flash chromatography (CH_2Cl_2/MeOH 9.5/0.5 0.2) to give colorless crystals (69%). HPLC: t_R = 15.68 (Column 1). MS (ESI): $[M + H]^+$ = 414.

In vitro release studies: In vitro release studies were performed by dialysis using a dialysis membranes (Merck Millipore, Milan, Italy) with 15,000 Da cut-off. Sink conditions and QSi solubilization were established adding 30% methanol by volume to the aqueous receiving phase [1]. Then, 20 mL of receiving phase were poured in the external compartment and continuously stirred at 500 rpm with a magnetic bar. Then, 2 mL of each QSi-loaded LP-SA were placed in the dialysis bag then at predetermined time intervals 0.15 mL of receiving phase were withdrawn and QSi content was evaluated by HPLC as above reported. Each removed sample volume was replaced with the same amount of fresh receiving phase. The obtained results are reported in Figure S1.

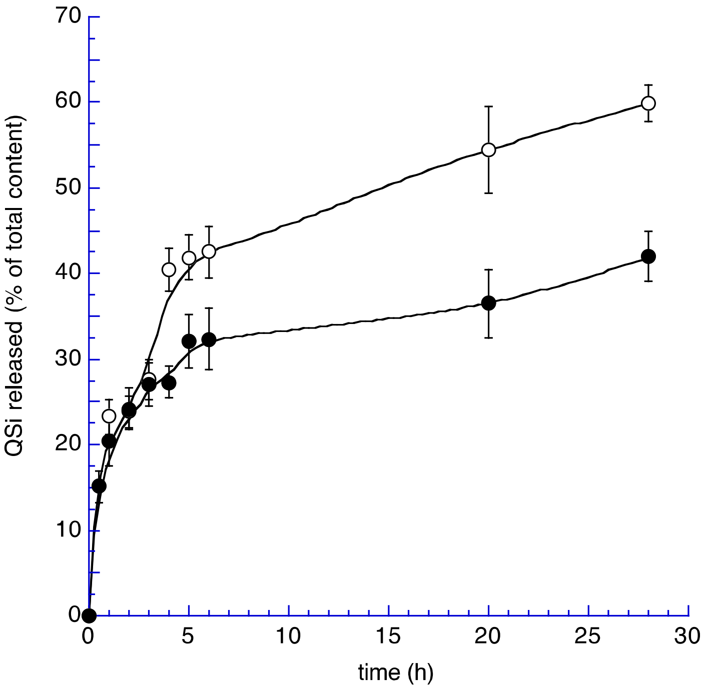

Figure S1. Release kinetics of CDC (○) and PF (●) from LP-SA, as determined by dialysis. Data are the mean of three experiments ± s.d.

Reference

1. Siewert, M.; Dressman, J.; Brown, C.K.; Shah, V.P. FIP/AAPS guidelines to dissolution/in vitro release testing of novel/special dosage forms. *Aaps Pharmscitech* **2003**, *4*, E7, doi:10.1208/pt040107.
